# Supplementary material for: The Impact of Point-of-Care Blood C-Reactive Protein Testing on Prescribing Antibiotics in Out-of-Hours Primary Care: A Mixed Methods Evaluation
Source: Antibiotics (Basel). 2022 Jul 26;11(8):1008. doi: 10.3390/antibiotics11081008 (PMC9332095; doi:10.3390/antibiotics11081008)
Supplement: Supplementary file 1 [file antibiotics-11-01008-s001.zip › antibiotics-1804379-supplementary.pdf]

Supplementary Material

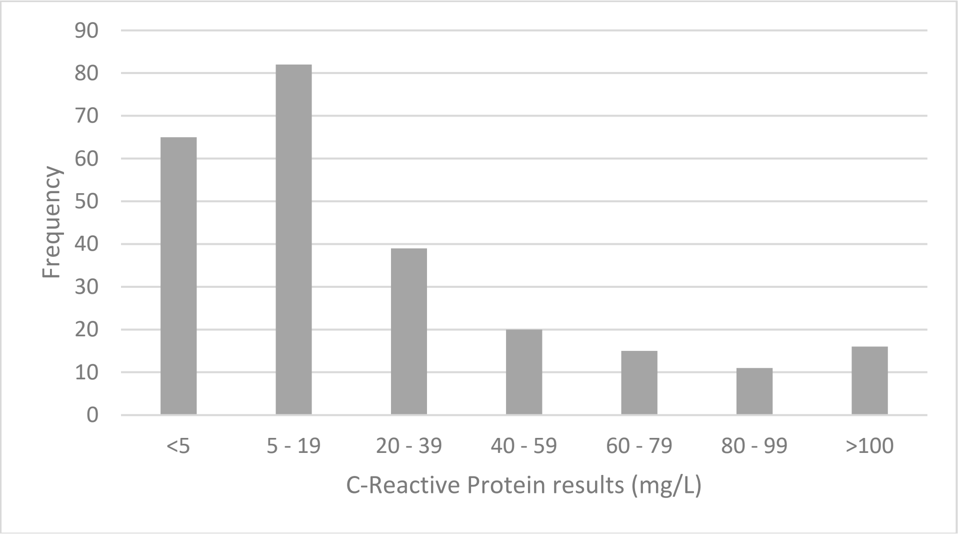

Supplementary Figure S1. C-Reactive Protein values from OOH care using Afinion 2 machines.

Clinician	Total tests	Tests at Site A	Tests at Site B	Tests at Site C
1	44	44	0	0
2	37	37	0	0
3	16	16	0	0
4	7	0	7	0
5	7	0	7	0
6	5	5	0	0
7	5	0	5	0
8	4	0	4	0
9	4	4	0	0
10	3	1	2	0
11	2	2	0	0
12	2	2	0	0
13	2	1	0	1
14	1	1	0	0
15	1	1	0	0
16	1	1	0	0
17	1	1	0	0
18	1	1	0	0
19	1	1	0	0
20	1	1	0	0
21	1	0	1	0
22	1	0	1	0
Illegible	5	5	0	0

Supplementary Table S1. Clinicians' test use by site.

Supplementary file S1

Based on the methodology proposed by *Fanshawe et al.* (2022), k time series forecasts for a period of interest t (say from $t=n+1\ldots n+k$) may be used to evaluate whether the expected (simulated forecasts) trend deviates significantly from the observed trend using Monte Carlo simulation and Wald Z statistics. First, the sum of observed time series values in the period following the intervention is computed ($\sum_{t=n+1}^{n+k} y_t$). Based on the fitted ARIMA model, several realisations ($n_{sims} = 1000$) of forecasted values, $\widehat{y_t}$, are estimated and their means and standard deviations of the forecasted totals computed as $\widehat{m_k} = \frac{\sum_{t=n+1}^{n+k} \widehat{y_t}}{n_{sims}}$ and $\widehat{s_k}$ respectively.

Thereafter the Z statistics can be computed for each location and globally (overall) by utilising the formulae $\frac{\sum_{t=n+1}^{n+k} y_t - \widehat{m_k}}{\widehat{s_k}}$ and $\frac{Z_I - Z_c}{\sqrt{\frac{1}{n}+\frac{1}{m}}}$ respectively. Note that Z_I and Z_c represent the mean Z statistics across the intervention and non-intervention locations whereas n and m are the number of locations classified as intervention and non-intervention respectively.

Location	Observed sum	Predicted sum	Standard deviation of predicted sum	Location Z statistic	Global Z statistic
A	4629	5369.9879	195.7385	-3.7856	
B	4210	3966.9963	208.2627	1.1668	
C	4764	5118.8087	605.2857	-0.5862	
D	4570	4678.0159	289.3443	-0.3733	-0.1291
E	4584	4447.1928	288.2058	0.4747	
F	1728	2234.5148	140.0560	-3.6165	
G	3761	3814.9759	148.3847	-0.3638	

Supplementary Table S2*:* The results showing the Z statistics testing the difference in trend between the observed and forecasted values of all antibiotics prescribed for adult patients by location and overall. The highlighted rows show results from locations with allocated POC CRP machines.

Supplementary Table S2 shows that for total antibiotics prescriptions considered, there were two locations in which the forecasted trend differed significantly from the observed trend with Z value being larger than the ± 1.96 (the critical values from the standard normal distribution for $\alpha = 5\%$). In these locations (one with POC CRP machine allocated, one without), the forecasted mean trends were of higher magnitudes than the observed values. In addition, overall, there were no statistical differences in trend between the forecasted mean values and the observed values between locations with POC CRP machines and those without, we can therefore conclude (as was also seen from the graphs in the result section) that POC CRP machines did not result in a significant reduction in all or chest antibiotic prescribing in the locations studied over the period of interest.

Location	Observed sum	Predicted sum	Standard deviation of predicted sum	Location Z statistic	Global Z statistic
A	1966	2109.8674	107.2798	-1.3410	
B	1715	1498.3183	143.5868	1.5091	
C	1972	2052.3930	292.9684	-0.2744	
D	1526	1470.7992	168.1690	0.3282	0.5408
E	1898	1621.9023	172.6338	1.5993	
F	826	1065.8755	92.4581	-2.5944	
G	1797	1916.4588	105.9880	-1.1271	

Supplementary Table S3: The results showing the Z statistics testing the difference in trend between the observed and forecasted values of chest antibiotics prescribed for adult patients by location and overall. The highlighted rows show results from locations with allocated POC CRP machines.

Antibiotic
Amoxicillin
Amoxil
Augmentin
Augmentin-duo
Azithromycin
Cefalexin
Clarithromycin
Co-amoxiclav
Doxycycline
Erythrocin
Erythrolar
Erythromycin
Erythroped
Oxytetracycline
Tetracycline

Supplementary Table S4. List of respiratory tract targeted antibiotics (initially obtained using the British National Formulary in conjunction with prescription lists from the observed data files, and reviewed by practising general practitioners who had access to local and national antimicrobial prescribing guidelines):

Supplementary file S2

CRP Blood testing in OOH Primary care bases
Topic guide for clinician interviews

General questions about Point of Care Testing POCT

- What(if any) is your previous experience or knowledge of POCT (any test)?

- What (if any) ideas/beliefs/attitudes did you have towards POCT testing before you first used it?
- If no experience, what are ideas/beliefs/attitudes about using POCT (any test)?

Questions specific to use of CRP

- What are your thoughts about using CRP blood test?
- What are your thoughts about having the CRP test available ?
- If you have used it, can you tell me what happened when you did? Can you also tell me about a situation when you chose not to use the CRP test?
- Why did you choose to do the CRP test?
- When would you think a CRP would be useful ? when might is not be helpful? are there any situations where you would definitely want to use a CRP test ? any situations where you might not ?
- What would you do if the CRP (done by you or a colleague) did not "fit" with your clinical assessment ? for example, if high in a patient who looked "well", or was low in a patient who you were concerned about?
- Would you want to be able to use CRP testing in children presenting to out of hours?
- Did you use any other tests? Are there other tests you would like to have had access to? or not? Tell me more.
- How did using the CRP test influence your decision making (NB either being able to do the test OR the results of the tests) – or not?
- What did you do following on from the test result?
- What did you tell the patient about doing the test?
- What did you think the patient's/carer's perception was of you doing the test?
- How did you find communicating re: this? Did doing the test make it easier or harder to make a plan/discuss this with the patient?
- How did you find doing the test practically? Probe ease/practical considerations/equipment?
- Was there anything you would like to have known before doing the test?
- How do you feel about using this test (or any others you have access to) in OOH now?
- Would you do the same actions again in a similar situation?
- Do you think having access to CRP makes the job easier or harder?
- Do you have any other thought or reflections?
